# Collectivism Is Associated With Greater Neurocognitive Fluency in Older Adults

**DOI:** 10.3389/fnhum.2019.00122

**Published:** 2019-04-11

**Authors:** Luis D. Medina, Melody Sadler, May Yeh, J. Vincent Filoteo, Steven Paul Woods, Paul E. Gilbert

**Affiliations:** ^1^Department of Psychology, University of Houston, Houston, TX, United States; ^2^Department of Psychology, San Diego State University, San Diego, CA, United States; ^3^Department of Psychiatry, University of California, San Diego, San Diego CA, United States

**Keywords:** executive function, cognition, culture, self-construal, verbal fluency

## Abstract

Neuropsychological research has been limited in the representation of cultural diversity due to various issues, raising questions regarding the applicability of findings to diverse populations. Nonetheless, culture-dependent differences in fundamental psychological processes have been demonstrated. One of the most basic of these, self-construal (individualism, collectivism), is central to how many other differences are interpreted. Self-construals may have possible consequences on social interactions, emotions, motivation, and cognition. This study aimed to evaluate the impact of self-construal on neurocognitive functions in older adults. A total of 86 community-dwelling older adults 60 years and older were assessed with three common self-report measures of self-construal along individualism and collectivism (IC). A cognitive battery was administered to assess verbal and non-verbal fluency abilities. Latent profile analysis (LPA) was used to categorize individuals according to IC, and one-way analyses of covariance (ANCOVA), including relevant covariates (e.g., ethnicity, gender, linguistic abilities), were used to compare neurocognitive functions between individualists and collectivists. Collectivists outperformed individualists on left frontally-mediated measures of verbal fluency (action, phonemic) after controlling for relevant covariates, *F*_(1,77)_ = 6.942, *p* = 0.010, *η*^2^ = 0.061. Groups did not differ on semantic fluency, non-verbal fluency, or attention/working memory (all *p*s > 0.05). These findings suggest a cognitive advantage in collectivists for verbal processing speed with an additional contribution of left frontal processes involved in lexicosemantic retrieval. Self-construal may provide a meaningful descriptor for diverse samples in neuropsychological research and may help explain other cross-cultural differences.

## Introduction

As the population ages, cultural diversity continues to change and grow at both a nationwide and global scale. Cultural diversity relates to both internal and external factors. Kitayama and Park (2010) explain that culture has three main constituents: explicit values, cultural tasks intended to achieve the culture’s primary values, and the implicit psychological and neural tendencies aligning with those values. It is theorized that both micro (biological) and macro (behavioral) aspects of culture are associated with brain processes that change as a function of an individual’s engagement in culture-specific ideas and practices, which supports the notion that there are dynamic neuro-cultural interactions (Kitayama and Uskul, 2011). In other words, given that culture involves explicit behaviors and processes, synchronous firing of neurons during cultural tasks results in those neurons being wired together; therefore, cultural tasks can shape and modify neural pathways (Kitayama and Park, 2010).

In studying neuro-cultural interactions, the use of constructs such as race, ethnicity, nationality, and other related demographic variables have complicated research. Some argue that this type of categorizing lends itself to seeing culture-specific, “emic” (i.e., having to do with internal elements of a specific culture, ignoring cross-cultural schema) attributes as differentiating members of contrasting cultures, rather than using a more “pan-cultural” (i.e., having to do with culturally universal elements) approach (Bochner, 1994) that allows for the clustering of cultures based on an underlying process that spans categories used in past research (i.e., race, ethnicity, or nationality). Others have argued racial categories lack conceptual meaning when scientific method principles are applied (Helms et al., 2005). Specifically, racial categories are associated with a complex network of factors, such as biological (e.g., skin color) and social/socio-political factors (e.g., the experience of racism), that may limit interpretation of findings (e.g., why a racial group performs lower on cognitive tests than another). Most often, research that attempts to report or account for cultural diversity utilizes predetermined “check-box” classifications, such as race or ethnicity, where an individual is asked to check off a box that most closely describes how the person identifies him-/herself. Additionally, such labels create clear distinctions that do not account for individuals who effectively blur the lines between categories (e.g., individuals with dual-citizenship, persons who identify as being members of more than one group; Arnett, 2002). These methods of narrowly categorizing individuals may also impact statistical power to make inferences.

In light of the limitations of demographic variables like race/ethnicity, other cultural factors may provide added benefit when examining neuro-cultural interactions. One of the most basic cultural dimensions, how people perceive the self, has been central to how many culture-dependent differences are interpreted and explained. Described as construals of the self, or self-construals, that are either independent (or *individualistic*) or interdependent (or *collectivistic*), the development of self-perception can be traced to early childhood and parental rearing practices, then further reinforced by peers and society. Self-construals of individualism or collectivism (IC) have been implied as having possible consequences not only on social roles and interactions, but also on emotions, motivation, and cognition (Markus and Kitayama, 1991). Much of the research on self-construal and relevant cultural differences has relied primarily on an “East-West” paradigm. Western cultures (e.g., United States), described as highly individualistic, tend to place greater value on the personal self, applying a schema of independence to social perception, and grounding their emotional life and motivation primarily on personal goals, desires, and needs. Eastern cultures (e.g., Japan), described as highly collectivistic, place greater value on their interpersonal self, applying this schema to their social perception, and grounding their emotions and motivations largely on social goals and concerns (Kitayama and Park, 2010).

The individual development of cognition takes place in a cultural context. Self-construals may pull cognitive resources differently, and culture-dependent meaning systems may then alter neural processing (Chiao and Ambady, 2007). Studies examining IC-related group differences on functional magnetic resonance imaging (fMRI) have suggested differential cortical representation and functional distinctions in several frontal lobe regions, such as the medial prefrontal cortex (Chiao et al., 2010). This evidence supports the notion that self-construal is a higher order function potentially mediated by frontal networks. Of note, collectivism has recently been associated with a reduced orbitofrontal cortical volume (Kitayama et al., 2017), suggesting self-construal may interact with neuroanatomical changes in the frontal lobe that may be relevant to cognitive processes.

This role of culture on frontal lobe processes has been supported by cognitive research. There is evidence that collectivist children outperform individualists on various inhibition, card sorting, and tower-building tasks believed to be related to these higher order cortical processes (e.g., Sabbagh et al., 2006). In an adult sample, Cagigas (2008) reported evidence that cultural differences on cognitive measures may be more attributable to higher cortical functions than more primitive subcortical systems. Coupled with the evidence from neuroimaging research, an argument can be made that behavioral manifestations of frontal-lobe dependent cognitive processes exist between collectivists and individualists.

Developmentally, it is not clearly understood when in the lifespan particular cross-cultural cognitive differences begin to emerge (Kitayama and Uskul, 2011); as described above, differences are observed in young children. However, given gray and white matter changes that largely begin to occur later in life around middle age (Bartzokis, 2004), it is likely that the aging process further impacts cross-cultural differences in cognition. Although age-related changes in cognition have been extensively documented, little research has examined the impact of age in the context of self-construal and cognition. Aging represents the consequences of biological processes while culture represents sustaining experiences and their effects (Na et al., 2017). Age and self-construal may interact in a manner that impacts personal relevance and, therefore, cognition and processing of information. Indeed, citing other studies examining the activation of object-processing areas on fMRI and demonstrating age-by-culture interaction, it has been suggested that culture may modulate neurocognitive aging (Park and Gutchess, 2006). However, most evidence of cultural effects on neural function in the context of the aging brain has been in perceptual processing (Park and Huang, 2010).

In healthy aging, psychophysiological studies have shown a frontal phenomenon in cognitive processing. Age-related differences in frontal networks crucial for attention and executive functions (EFs) have been shown in brain imaging studies (e.g., McGinnis et al., 2011). While deterioration of both gray matter and white matter are observed in healthy aging, some researchers now argue that cognition is associated more with white matter changes than with cortical thickness (Ziegler et al., 2010), and EF and processing abilities have been explained as the primary cognitive domain affected by white matter alterations (Murray et al., 2010). In light of the aforementioned implications of culture and higher cortical functions associated with the frontal lobe, age-related differences in frontal networks—both in gray and white matter—may interact with culture in a manner that impacts executive functioning abilities.

EFs are a multi-faceted construct generally understood as complex higher mental processes used in goal-setting, planning, and execution of plans (Lezak, 1982). One EF involves the production of intended actions while self-regulating through the inhibition of unrelated or irrelevant actions (Lezak et al., 2004), herein referred to as *cognitive fluency*. Neuropsychologically, cognitive fluency is often evaluated *via* measures where one is asked to rapidly generate a series of novel responses within a category or a set of rules and within a time limit (e.g., a minute).

Cognitive fluency tasks have been shown to be particularly sensitive to frontal lobe integrity (Baldo et al., 2001). This sensitivity may be due in part to the various components of fluency measures, such as semantic and inhibitory processes associated with cortical gray matter integrity (McDowd et al., 2011) as well as the speed of response and mental organization associated with white matter integrity (Kempler et al., 1998). Given the effects of aging on these areas, it is not surprising that performance on fluency measures is generally worse in older adults than in young adults (Elgamal et al., 2011).

The current study attempted to expand on the dearth of research by examining the relationship of IC with cognitive processes sensitive to frontal lobe functioning in an older population. As suggested by extant literature, self-construal may moderate age-related frontal lobe changes and, therefore, cognitive processes associated with frontal lobe integrity. Given the nature of collectivism and its prioritization of one’s social group over the individual as well as the impact of aging on frontal lobe networks, we hypothesized that cognitive burden would be greater in this group. More specifically, we predicted that collectivism would be associated with greater demands on executive functioning and frontal lobe function, reducing the availability of frontally-mediated resources necessary to perform well on EF-dependent tasks. Therefore, it was expected that collectivists would perform lower than individualists on cognitive fluency measures. Additionally, it was expected that both groups would perform similarly on measures not related to cognitive fluency.

## Materials and Methods

### Participants

Individuals at least 60 years old were recruited from the general community in San Diego, CA, USA using fliers and contacts with community programs serving older adults. Individuals who reported not feeling comfortable reading, writing, and speaking in English enough to complete study measures were excluded. Participants had to be community-dwelling adults able to provide written informed consent and not have endorsed a diagnosis of dementia or another cognitive disorder (e.g., mild cognitive impairment) at the time of testing. Although participants were not excluded from participation based on the history of psychiatric diagnoses or substance use, information regarding these factors was collected to control for their potential effect on performance in data analysis. Additionally, the Mini-Mental State Exam (MMSE; Folstein et al., 1975) was administered to participants to offer a brief screen for possible cognitive impairment associated with aging. Individuals with MMSE scores below 26 were not included in the data analysis. A total of 100 individuals were recruited. *A priori* standard power calculations indicated this sample size would be adequately powered to detect medium-to-large effects with an alpha level of 0.05.

All participants signed written informed consent approved by the Institutional Review Board at San Diego State University and were tested in person using paper-and-pencil versions of the measures described below. Testing was completed by a trained psychometrist in a quiet room, free of distraction, and took place either in a laboratory setting or in the community at a companion senior day center site. Testing lasted for approximately 1 h and participants received $10 for their participation.

### Measures

#### Demographic

Participants were administered a semi-structured interview regarding their demographic background (e.g., birthplace, race and ethnicity, first language spoken, fluency in the English language, years living in the United States, acculturation) as well as pertinent medical history (e.g., history of head injury, learning disability, stroke, substance use). Validated self-report measures were used to assess bilingualism and acculturation. Given that the interview and test battery were administered in English, bilingualism and English-language dominance have implications on participants’ responses and performance. Acculturation, while a construct dissociable and discrete from self-construal, may have implications on self-construal. An individual who is more acculturated to the dominant culture of where the individual resides may adopt values related to the self-construal typically observed within that culture (e.g., a person from a collectivistic culture, like Japan, who immigrates and acculturates to a “Western” culture, like the United States, may become more individualistic). For bilingualism, the Bilingualism Dominance Scale (BDS; Dunn and Fox Tree, 2009) was used. This brief scale is administered as an interview and consists of 12 items, which evaluate the person’s predominant use of one language over another or the equal use of the two languages by targeting three main criteria: percent of language use, age of acquisition, and restructuring of language fluency. Original validation studies of the BDS showed it significantly predicted respondents’ scores on objective measures of verbal fluency and translation reaction times. The Stephenson Multigroup Acculturation Scale (SMAS; Stephenson, 2000) was used to assess acculturation. This reliable and validated self-report measure of acculturation consists of 32 statements rated on a four-point scale (i.e., false, partly false, partly true, true). Items relate to domains of language, interaction, media, and food in the context of either the society of origin or the current society of residency. The measure allows for the calculation of two indices: the dominant society immersion (DSI) and the ethnic society immersion (ESI). Original validation studies of the SMAS showed high internal consistency for the entire scale (coefficient α = 0.86) and for each of the indices (DSI = 0.97; ESI = 0.90).

#### Self-Construal

In a review of various survey methods, Peng et al. (1997) found significant limitations of rating and ranking measures in the assessment of self-construal when used to differentiate between cultures. Due to the unsatisfactory limitations of these methods, this study used a mixed methods approach using three measures validated for this purpose: INDCOL (Triandis, 1996), Scenarios (Triandis and Gelfand, 1998), and the Twenty Statements Test (TST; Kuhn and McPartland, 1954). Responses for the TST were coded by two raters blind to the participant’s responses on other test materials, including other self-construal measures, and following the methods described elsewhere (Santamaría et al., 2010). Briefly, for purposes of determining self-construal, TST responses were coded along the domain of “organization” and statements were coded as either *private* (e.g., “I am smart”), *collective* (e.g., “I am a student”), or *public* (e.g., “I am someone who cares for others”). Raters were trained by the lead author on unrelated samples of TST responses that were not included in the current analyses. Once raters reached criterion (minimum Cronbach’s alpha of 0.85), they began rating of TST responses from the recruited sample and were blind to participants’ responses to the other measures of self-construal. Discrepant scores were regularly discussed with the lead author but were not removed from analyses. Inter-rater reliability was assessed using the intra-class correlation coefficient (ICC) and was found to be high (Cronbach’s alpha = 0.97, ICC = 0.94).

#### Cognitive

The cognitive battery largely assessed for cognitive fluency as well as attention and working memory. Included in the battery were several tests. In the Action Fluency Test (Piatt et al., 1999), individuals name as many verbs as they can in a minute’s time. Similarly, in phonemic letter fluency with FAS, respondents provide words that begin with the letters F, A, and S within a minute for each letter. In semantic fluency, responses are elicited for particular categories; animals and vegetables were used for the current study and each category was given a minute for responses. The Design Fluency subtest of the Delis-Kaplan Executive Function System (D-KEFS; Delis et al., 2001) is a nonverbal measure in which respondents make line designs following specified rules within a minute. Attention and working memory abilities were assessed with the forward and backward conditions of Digit Span. These measures were chosen and categorized based on the neuroanatomical correlates they have been shown to represent. Verbal fluency measures like Action Fluency and FAS engage more left frontal lobe regions while semantic fluency (e.g., animals, vegetables), which relies more on conceptual knowledge, engages slightly more posterior regions (i.e., frontal-temporal areas) in the left hemisphere; in contrast, D-KEFS Design Fluency appears to engage right hemisphere frontal lobe regions in an analogous fashion to measures like FAS (Lezak et al., 2012). Measures of attention and working memory like Digit Span have shown evidence of broader, more diffuse engagement of brain regions; in addition to the bilateral involvement of dorsolateral prefrontal cortex, Digit Span performance appears to involve bilateral occipital and parietal areas that suggest use of visual imagery strategies during the task (Gerton et al., 2004). Reading ability in English was assessed with the Reading subtest of the Wide Range Achievement Test—Fourth Edition (WRAT-4; Wilkinson and Robertson, 2006). The WRAT-4 Reading has been shown useful in estimating premorbid ability and serving as a proxy of education quality in English, particularly in ethnically diverse samples with heterogeneous educational backgrounds (Manly et al., 2005).

#### Mood

Mood was assessed for each participant using the Geriatric Depression Scale (GDS; Yesavage et al., 1982), a validated measure of depression, to control for the possible effect of mood on cognition. Mood was then considered as a potential covariate and included in analyses as necessary.

### Analysis

#### Self-Construal

Given the number of variables provided by the self-construal measures, latent profile analysis (LPA) was used to identify typologies of people, as opposed to a taxonomy of variables, along the lines of IC using the multiple variables provided by the self-construal measures. LPA is a person-centered technique in which an individual can be assigned to a mutually exclusive profile based on that individual’s responses to observed continuous variables of interest by maximizing homogeneity within groups and maximizing heterogeneity between groups (Roesch et al., 2010). The process of LPA seeks to reveal the underlying latent construct of responses/scores (Lanza et al., 2003). Various models are tested to determine the optimal number of profiles and the best-fitting model is chosen based on various statistical indices of fit. IC measures provided a total of seven scores per participant that were included in the LPA. A 2-, 3-, and 4-profile solution were tested. By capitalizing on the shared variance of all the self-construal measures, LPA would allow for a more reliable, “error-free” (Roesch et al., 2010) categorization of individuals than would be achieved by using only a single measure.

#### Covariates

Relevant covariates were chosen for theoretical and statistical reasons. Covariates of interest included: ethnicity (Hispanic, non-Hispanic), race (White, non-White), age, gender, self-reported English fluency, WRAT-4, years in the United States, age when moved to the United States, bilingualism, acculturation, and mood (GDS). Univariable analyses were used to determine significant covariates. Cognitive domains were regressed on covariates of interest. Variables significant at a *p* ≤ 0.10 threshold were included as covariates in subsequent analyses.

#### Cognition

For data reduction purposes, composite scores were created such that cognitive measures of interest were grouped into four domains: Verbal Fluency (Action Fluency Test, FAS), Semantic Fluency (Animals, Vegetables), Nonverbal Fluency (Design Fluency Conditions 1 and 3), and Attention/Working Memory (Digit Span Forward and Backward). One-way analysis of covariance (ANCOVA) was used to examine the relationship of self-construal on cognitive domains. Data were checked for normality and outliers and missing data were excluded from analyses. Findings with *p*-values at or less than 0.05 were considered significant.

## Results

Of the 100 participants recruited, six individuals withdrew consent or were unable to complete all measures, seven were excluded from analyses due to MMSE scores less than 26, and one was excluded due to outlying values (>2 standard deviations) on several cognitive measures in addition to a history of head injury and self-reported cognitive symptoms. Therefore, 86 individuals (age: 67.2 ± 6.0; education: 14.5 ± 2.8) were included in the final analyses (Table 1).

**Table 1:** Sample demographics.

	Sample *N* = 86		Individualists *N* = 42		Collectivists *N* = 44		*p*
	Range	Mean (SD)	Range	Mean (SD)	Range	Mean (SD)	
Age (years)	60–88	67.2 (6.0)	60–88	66.9 (6.8)	60–78	67.6 (5.2)	0.600
Education (years)	8–20	14.5 (2.8)	8–20	15.0 (3.03)	8–20	14.0 (2.6)	0.100
Years in US	4–80	61.5 (14.5)	21–80	62.5 (10.7)	4–78	60.5 (17.5)	0.533
WRAT-IV	39–76	59.4 (7.9)	40–70	61.6 (6.8)	39–76	57.4 (8.5)	0.014
GDS	0–13	2.8 (3.2)	0–13	3.1 (3.7)	0–10	2.5 (2.7)	0.399
SMAS	−1.2 to 2.3	0.1 (0.5)	−0.6 to 2.3	0.2 (0.6)	−1.2 to 1.9	0.1 (0.5)	0.327
BDS—English	6–26	24.4 (4.1)	6–26	25.0 (3.7)	7–26	23.9 (4.5)	0.232
BDS—Other	−2 to 27	2.8 (6.3)	−2 to 27	1.7 (5.1)	−2 to 22	3.8 (7.1)	0.134
	***n***	***%***	***n***	***%***	***n***	***%***	***p***
**Gender**							
Male	40	46.5	22	52.4	18	40.9	0.387
Female	46	53.5	20	47.6	26	59.1	
**Ethnicity**							
Hispanic	6	7.0	2	4.8	4	9.1	0.677
Non-Hispanic	77	89.5	38	90.5	39	88.6	
**Race**							
American Indian	1	1.2	0	0	1	2.3	0.300
Asian	5	5.8	1	2.4	4	9.1	
African-American/Black	11	12.8	4	9.5	7	15.9	
Multiple	4	4.7	1	2.4	3	6.8	
Pacific Islander	1	1.2	0	0	1	2.3	
Unknown	3	3.5	1	2.4	2	4.5	
Caucasian/White	61	70.9	35	83.3	26	59.1	
**Birthplace**							
United States	74	86.0	38	90.5	36	81.8	0.213
Other	12	14.0	4	9.5	8	18.2	

*Note: WRAT-IV, Wide Range Achievement Test—Fourth Edition, Reading Score; GDS, Geriatric Depression Scale (higher scores are associated with greater depressive symptoms); SMAS, Stephenson Multigroup Acculturation Scale (higher scores are associated with greater immersion in dominant society); BDS, Bilingual Dominance Scale (higher scores are associated with greater reliance on that language). “Other” birthplaces included Chile (1), Colombia (1), Iraq (1), Latvia (1), Philippines (4), Sweden (1), Switzerland (1), and Vietnam (1). For those born outside the US, self-reported years in the US ranged from 4 to 80 years (mean = 36.1 ± 22.1) and years speaking English ranged from 8 to 80 years (mean = 50.8 ± 18.8). Significance values were calculated using *t*-tests for Age, Education, Years in US, WRAT-IV, GDS, SMAS, and BDS; Fisher’s exact test for Gender and Ethnicity; and Chi-square test for Race and Birthplace*.

### LPA

Model fit indices for the LPA did not indicate a significant improvement of the 3- and 4-class solutions. Therefore, the more parsimonious 2-class solution was considered a better fit to the data (see Lubke and Muthén, 2005). Classification in LPA is based on the probabilities of being within a class/profile, which are related to the means of the individual indicators. A qualitative review of the 2-class solution (Table 2) revealed one profile (Class 1) consistent with higher scores on individualism scales compared to collectivism scales; therefore, Class 1 was labeled “individualists.” Similarly, the profile observed in Class 2 was consistent with higher scores on collectivism scales relative to individualism scales and was labeled “collectivists” as a result. LPA results identified 42 individualists and 44 collectivists. Self-construal groups did not significantly differ on demographic variables, including gender, ethnicity, or race (Table 1).

**Table 2:** Means and standard deviations (SD) of self-construal measures.

Measure	Sample *N* = 86		Individualist *N* = 42		Collectivist *N* = 44	
	Range	Mean (SD)	Range	Mean (SD)	Range	Mean (SD)
IND	3.2–7.8	5.8 (1.1)	3.3–7.8	6.0 (1.1)	3.2–7.3	5.5 (1.1)
COL	3.1–9.0	6.8 (1.3)	3.1–8.5	6.2 (1.3)	5.1–9.0	7.3 (1.0)
Scenarios—Individualism	3.0–13.0	8.3 (2.4)	9.0–13.0	10.3 (1.4)	3.0–8.0	6.4 (1.5)
Scenarios—Collectivism	1.0–13.0	7.5 (2.5)	1.0–7.0	5.5 (1.5)	6.0–13.0	9.4 (1.5)
TST—Private	0.0–7.0	3.3 (2.2)	0.0–7.0	3.6 (2.2)	0.0–7.0	3.0 (2.1)
TST—Collective	0.0–7.0	2.4 (2.0)	0.0–7.0	2.1 (2.1)	0.0–6.5	2.7 (1.9)
TST—Public	0.0–4.0	0.9 (1.0)	0.0–4.0	0.9 (1.1)	0.0–4.0	0.9 (1.0)

*Note: IND, INDCOL mean individualism score; COL, INDCOL mean collectivism score; TST, Twenty Statements Test*.

### Covariates

Univariable analyses with a *p* ≤ 0.10 threshold determined inclusion of only four covariates: ethnicity, gender, GDS, and WRAT-4 Reading.

### Cognition

**Figure 1:**
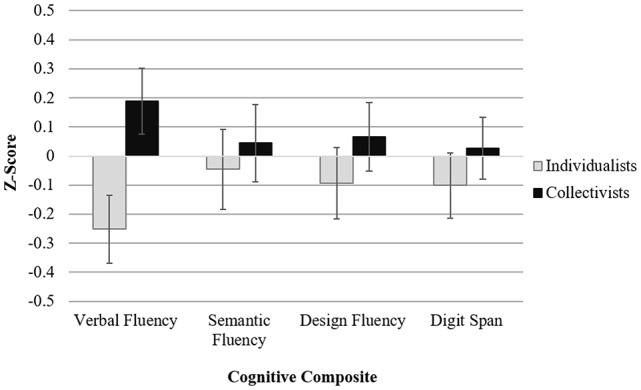
Estimated means (SE) on cognitive measures by self-construal controlling for ethnicity, gender, Geriatric Depression Scale (GDS) score, and Wide Range Achievement Test (WRAT)-Reading score.

ANCOVA controlling for the determined covariates revealed a medium-sized main effect of self-construal on Verbal Fluency (Action Fluency Test, FAS), *F*_(1,77)_ = 6.942, *p* = 0.010, η^2^ = 0.061. Comparing the estimated marginal means showed that collectivists (*M* = 0.189, SE = 0.113) outperformed individualists (*M* = −0.252, SE = 0.117). No significant differences (all *p*s > 0.05) were noted on Semantic Fluency (Animals, Vegetables), or Nonverbal Fluency (Design Fluency, both conditions). Both groups performed similarly (all *p*s > 0.05) on measures of Attention/Working Memory (Digit Span forward and backward; see Figure 1). The estimated means for individual tests included in composite scores are shown in Table 3.

**Table 3:** Estimated means (SE) on cognitive measures by self-construal controlling for ethnicity, race, and WRAT-Reading.

Measure	Individualist *N* = 42			Collectivist *N* = 44		
	Mean	SE	95% CI	Mean	SE	95% CI
AFT	21.94	0.83	20.3–23.6	23.12	0.80	21.5–24.7
FAS	32.03	1.72	28.6–35.4	37.78	1.66	34.5–41.1
Animals	18.01	0.71	16.6–19.4	18.94	0.68	17.6–20.3
Vegetables	11.54	0.60	10.4–12.7	12.15	0.58	11.0–13.3
Design fluency switch	6.03	0.25	5.3–6.7	6.19	0.33	5.5–6.9
Design fluency non-switching	8.59	0.45	7.7–9.5	9.00	0.43	8.1–9.9
DS-F	9.63	0.29	9.1–10.2	9.81	0.28	9.3–10.4
DS-B	6.62	0.30	6.0–7.2	6.68	0.29	6.1–7.3

*Note: SE, standard error; CI, confidence interval; AFT, Action Fluency Test; FAS, phonemic fluency with F, A, and S; DS-F, digit span forward; DS-B, digit span backward*.

## Discussion

In the current study, we examined the role of self-construal on frontal lobe-mediated processes. Given the cognitive demands associated with collectivism, which involves the consistent perception of self in the context of others, we hypothesized that collectivists would perform worse than individualists on measures of cognitive fluency. These measures were used as they are more focally sensitive to frontal lobe changes associated with the aging process. Along these lines, we expected both groups to perform similarly on non-fluency measures (i.e., attention/working memory as measured by Digit Span forward and backward), which rely on more diffuse brain regions. Contrary to our expectations, collectivists performed significantly better on measures of verbal fluency (phonemic, action) after controlling for ethnicity, gender, mood, and linguistic ability. Both groups performed similarly on semantic and nonverbal fluency as well as on tests of attention/working memory. These findings support previous literature suggesting that self-construal may be differentially associated with verbal and nonverbal measures (Hedden et al., 2002). Notably, race and ethnicity did not differ between groups, possibly highlighting the utility of measuring self-construal as an underlying perceptual process in cross-cultural research.

As described previously, verbal fluency measures rely on several processes linked to various cortical regions. Interestingly, performance on these measures does not always rely on semantic memory or vocabulary knowledge. McDowd et al. (2011) explain that overall performance on verbal fluency measures is most consistently predicted by the speed of processing with an additional, albeit secondary, contribution of executive processes. This synergistic effect of processing speed and executive functioning might explain why the effect was not found in untimed attention/working memory verbal tasks (Digit Span forward and backward) or in non-verbal design fluency, which relies primarily on motor planning and visual scanning rather than processing speed (Suchy et al., 2010). Future studies that include measures of processing speed are needed to further investigate this hypothesis.

Our results complement extant literature in various ways. These findings suggest a differential effect of IC on frontal lobe mediated cognition. In a young adult sample, our group previously reported no effect of self-construal on cognition in young adults (Medina et al., 2014). In light of the current results, an effect of age is suggested such that older collectivists may be more accustomed than individualists to such executive and processing demands and, thus, are more capable of compensating. Taken together with the results in young adults, it may be that normal cognitive decline as part of the aging process may magnify differences between collectivists and individualists. That is, aging may differentially impact individualists who may be less likely to compensate for executive declines as we observed in collectivists. Furthermore, our findings indicate that this cognitive advantage exists in verbal measures of both action and letter fluency, but is not evident in other measures of verbal and nonverbal fluency. While letter fluency also shares similar pathways with semantic fluency measures, it has been proposed that there is greater demand of initiation and maintenance of retrieval strategies in letter fluency, similar to action fluency, suggesting greater sensitivity to left frontal lobe function in letter fluency and action fluency than in tests of semantic fluency (Piatt et al., 1999).

The current findings additionally raise some questions regarding how self-construal can play a role in applied settings. For instance, demographic variables such as race and ethnicity are more commonly accounted for, or at least documented, in clinical settings and normative samples relative to IC. However, it is unknown how much of their effect on cognitive measures is potentially mediated or moderated by self-construal. Given the dynamic nature of culture, it is difficult to ascertain how these and other demographic variables interact to paint an individual’s cognitive profile. More research in this area could help answer some of these questions.

This study was limited by several factors. The resulting sample size of 86 individuals was adequately powered (96%) for large effect sizes, but less powered (*post hoc* = 65%) for medium effect sizes. As shown in Table 1, the sample was limited in its diversity, particularly in relation to ethnicity and race, variables typically examined in the context of cultural differences and potentially related to self-construal. This might explain why, contrary to expectations, race and ethnicity were not related to self-construal. Nevertheless, while many studies on self-construal employ an East/West paradigm to capitalize on cultural differences, the study of self-construal within a homogeneous sample in a single culture has been previously demonstrated. For instance, work in this area has examined self-construal in both a purely Japanese sample, reflecting an “East,” or collectivist, population (Kitayama et al., 2017), as well as in a purely United States sample, reflecting a “West,” or individualist, population. Moreover, the self-construal scores observed in the reported sample are consistent with other published work on this topic. Specifically, on the TST, individualists showed a higher proportion of “private” or self-attribute (i.e., individualist) responses compared to collectivists, 72% vs. 22%, respectively—in a pattern consistent with results reported by Markus and Kitayama (1991); the opposite pattern was observed for collectivist responses on the TST: collectivists = 64%, individualists = 15%. Individualists and collectivists also demonstrated significantly different vertical individualism and vertical collectivism sub-scores in the expected directions on the INDCOL, consistent with the differences reported by Singelis et al. (1995) in a more diverse sample. Therefore, we are confident that the observed effect is consistent with the literature on self-construal. We hypothesize that this effect would be greater when using traditional East/West paradigms. In the context of our sample’s limited ethno-racial diversity, our results further support the measure and study of self-construal as a variable separate from race and ethnicity.

The current study also had a limited cognitive battery. In spite of evidence that the measures administered are sensitive to cognitive decline in older adults (see Clark et al., 2012), it remains possible that not all these measures are sensitive enough to detect differences in a healthy sample of individualists and collectivists. A more expansive battery of tests that are more challenging or sensitive might aid future research. Cultural neuroscience research utilizing neuroimaging techniques such as EEG and fMRI has also elucidated how processes and brain activation patterns may differ between cultures while performance remains equivalent between groups (e.g., Kitayama and Park, 2010). Therefore, in a similar fashion, although individualists and collectivists may perform similarly on a cognitive task, differences in process may exist. Neuropsychological tests allowing for the measure of process approaches (e.g., serial clustering vs. semantic clustering in a verbal list-learning task, global vs. local attention to drawing sequence in a complex figure task) might aid in the identification of these.

Lastly, without true experimentation, it is not possible to posit any causality in the relationship between self-construal and cognition. Recent literature has demonstrated the benefits of being able to prime self-construal in individuals to investigate the impact of IC on psychological processes (Chiao et al., 2010) and that such priming can impact performance on a contextual memory task (Grossmann and Jowhari, 2017). The direct effect of IC could thus be examined in the context of cognitive fluency and other neuropsychological performance using a prime/no-prime experimental design.

Despite these limitations, these findings have implications for future research. Neuropsychological research methods typically focus on traditional demographics, primarily those including race, ethnicity, gender, and age. However, we still do not fully grasp the mechanism underlying the role of cultural variables in cognition (Manly, 2005). Likewise, we are limited in our understanding of how these interact with other cultural variables or how other variables are similarly related to cognitive processes. As has been proposed by Na et al. (2017), cognitive functioning and cultural values may interact with each other through moderated mediation processes to determine cognitive processes. A cultural neuroscience framework incorporating multiple factors—micro and macro, biological and behavioral, process and performance—would aid greatly in expanding our comprehension of our increasingly diverse world. This attention to issues related to cultural diversity in our studies is likely to clarify past and future research findings.

## Ethics Statement

This study was carried out in accordance with the recommendations of the Declaration of Helsinki and the American Psychological Association ethical standards with written informed consent from all subjects. All subjects gave written informed consent in accordance with the Declaration of Helsinki. The protocol was approved by the San Diego State University Institutional Review Board.

## Author Contributions

LM, MS, MY, JF, SW and PG all provided substantial contributions either to the conception and design of the study, analysis, and/or interpretation of the results. LM also participated in data acquisition (subject recruitment and cognitive evaluation). Additionally, all authors critically revisited the work, approved its final version for publishing, and agreed to be accountable for all aspects of such work.

## Conflict of Interest Statement

The authors declare that the research was conducted in the absence of any commercial or financial relationships that could be construed as a potential conflict of interest.
